# Patents and Patent Litigation of the Dental Profession past and Present

**Published:** 1911-11-15

**Authors:** J. N. Crouse

**Affiliations:** Chicago


### PATENTS AND PATENT LITIGATION OF THE DENTAL PROFESSION PAST AND PRESENT.

BY DR. J. N. CROUSE, CHICAGO.

Read before the Chicago Dental Society, September, 1911.

I come before you to discuss some very important questions relative to patents and patent litigation of the dental profession. A discussion along this line seems necessary at this time, because there are so many erroneous ideas in the minds of dentists on this subject. My past experience, I feel, places me in a position to speak with some degree of authority.

Among the questions which I will freely discuss tonight, are the following:

What is a process patent?

What constitutes a valid patent?

Should an individual dentist take out patents and enforce patent claims?

What is the duty of the Dental Protective Association of the United States toward patent claimants?

With these questions before you, I will now proceed with the discussion of our subject. Except it be the farmer, there is no group or body of men that have suffered more from patent abuse in the past than have the members of the dental profession. In order that you may understand and appreciate the truthfulness of this statement, it is necessary for me to give you some past history and describe the conditions surrounding us and the schemes for getting our earnings before the organization of the Dental Protective Association of the United States, in 1888.

In the year 1851, a patent was obtained by Charles Goodyear on the process of hardening or vulcanizing rubber. This patent was declared valid and was granted to the rubber company largely on its utility. The dental profession paid the royalty for the use of rubber for vulcanizing plates until this patent expired. Then the parties controlling this company took out another patent known as the Cumming's patent. This was nothing more or less than a patent on the different steps in making rubber plates with nothing new or original in it. A process patent pure and simple on which our profession had already paid royalties for the full term of the patent. Before attempting to collect royalty on this patent the rubber company secured a decision in the Federal court validating their claims. The validity of this patent was again tested in the Supreme court and it was proven that the rubber company had hired the attorneys on both sides, and this fact caused the Supreme court to remand this patent back to the lower courts for a re-hearing. An attempt was made to raise money to fight this in the lower courts, but the dental profession was so demoralized and the lack of unanimity in the profession was so great, that nothing was accomplished and the rubber company collected royalty for seventeen years, or the life of the patent. It is a matter of history that many dentists went through bankruptcy to avoid paying the back royalty, and substituted celluloid for rubber which in time proved a failure, and the practitioners of that period, some of whom are still living, paid the rubber company many millions of dollars and that on a patent which would not have been worth the paper on which it was printed if it had been properly contested.

After the expiration of the Cumming's patent, another corporation was formed known as the International Tooth Crown Company. The promoters of this company, seeing the favorable condition of the dental profession, they having been trained by the rubber company to pay royalty, and with the skill and experience which comes from long practice, were able to organize just the kind of a company to best secure the object desired and make the dentists do their bidding.

This company soon began giving the dentists the greatest annoyance possible. They had taken out and acquired patents—numbering from thirty-five to fifty— which covered all manner of operations and devices, making it impossible to practice dentistry without infringing many of their patented claims. And having in their employ the attorneys and former agents of the old Goodyear Dental Vulcanite Company, they were fully equipped to again yoke the dental profession and make them pay royalties in much greater amounts and by much more disgraceful methods than were practiced by the old rubber company. They were issuing licenses and bringing suits as early as 1886, and at this time had a number of dentists under injunction. Those securing these licenses were obliged to agree, in writing, to recognize the validity of all the patents owned and controlled by this corporation.

One of the various methods they adopted was to get clinicians appointed at society meetings to instruct and work up an interest in methods on which they held patents, also to employ instructors to go amongst the dentists and teach their patented methods. Then their agent following had more definite knowledge as to whom to approach as infringers. After securing as many licenses as possible in a town, the agent would, by way of reward to the victims, insert this notice in the local papers:—

DENTAL NOTICE.

This is to certify that__________office No.____________ street, is our only licensee, to make and to furnish to patients in the city of______________any tooth crowns or bridge-work, so-called, manufactured under the patents of the International Tooth Crown Company. Said patents are construed to cover the most practical forms of artificial dentures now commonly known as crown and bridge work.

All persons are hereby cautioned against obtaining any such artificial dentures from any dentist not licensed, as none are authorized except by written license of said company.

The full legal penalty will be promptly enforced against all dentists, as well as their patients, making unauthorized use of any such patented denture.

A reward will be paid by said company to persons furnishing it with any cases of bridge-work of one, two or more crowns or bridges made by any dentist not licensed by this company.

(Signed) INTERNATIONAL TOOTH CROWN CO.

By way of enforcing their scheme, suits were commenced against several of the prominent men in different cities. Before the Protective Association was organized a large number of suits had been begun. Nor was this all, as five or six other companies had been formed with numerous patents on various methods of practice. Suffice it to say that it appeared to me as though our peace, manhood and prosperity were threatened on all sides.

After much thought and planning the Dental Protective Association was first presented to the profession at a meeting of the Odontographic Society of Philadelphia, in December 1888.

It must be remembered in this connection that the unsuccessful attempts in former years to get united action on the part of the profession made it more difficult to form this association, and the lack of encouragement and the urgent advice given me by leading minds to abandon the scheme would have discouraged a less hopeful individual. However, I kept on working and pleading with the dental profession all over the United States, and at the end of eight years, I had succeeded in getting 6,000 dentists into the organization.

Long before this, however, the association had retained the services of Offield & Towle, a well-known firm of patent attorneys of Chicago, and had taken charge of the defense of different dentists, who had been sued by the hundreds all over the United States, and succeeded in getting their licenses revoked and stopped the paying of their license fees, as a large number of the dentists were paying enormous revenues to the Crown Company. In several instances the royalties paid were as high as $400.00 or $500.00 a year and in a few cases even more than that.

In a suit brought by the International Tooth Crown Company against Richmond and others the record shows that everything was shaped so that the decree would make the Low bridge patent the only legitimate patent and all other bridge patents infringements. This would have been hard to understand only for the fact that the International Tooth Crown Company had previously acquired the Low patent, consequently this patent was the chief one in contention, and the only one on which they had a court decree in its favor.

In order to get this decision of the Federal court, which had validated the Low bridge patent, reversed, we felt it necessary to take a large amount of testimony, which we did, taking the testimony of fifty-five witnesses, scattered all over the United States, from Maine to California, and all of these witnesses I saw in person and from them learned what they could give us in the way of testimony, before taking their depositions before a Master. In all instances, I was present at the taking of this testimony before it was presented to the Court. This testimony amounted to over 500 pages, closely written, and we succeeded in the end in getting the decision reversed and putting the Crown Company out of business.

Had the Dental Protective Association not been in existence and the individual dentist left to himself, he would have been at the mercy of these patent sharks and every man practicing dentistry today would be paying hundreds of dollars in royalty on the crown and bridge-work he is making.

The history of the Protective Association would hardly be complete without adding that the Crown Company, after the association had tied them up and cut off their revenue brought suit against me for damage to their business for $60,000.00 and six months' imprisonment. That move didn't scare me in the least, and I immediately issued another circular announcing my decision to continue my part. Some time after this a representative of the Crown Company came on to Chicago and tried to buy me by bribery. I did not set any price on myself, but they offered me a million dollars if I would let them win two or three suits and then advise the profession to settle.

Some years later when the fight seemed to be over, a member of the Protective Association in the East said he believed the suit could be won yet, if I quit. I said that I had quit as I had no more funds and the profession did not seem interested. This party took it for granted that I would not fight any further and Sheffield and his party got into Court and took testimony again and got the Court to reverse its decision and sustain the Low bridge patent.

I then issued a circular to this effect, that if the dental profession wanted me to do anything for them they must come forward and pay $10.00 for their membership fee and by this method I more than doubled the membership.

With these funds I went into Court again and proved that the Crown Company had hired the attorneys on both sides, and when this was shown the Court again reversed its decision, knocking out the Low bridge patent. It will interest you to know that a bill is now pending in Congress to have the Low bridge patent reinstated, but we will not discuss this tonight.

Having disposed of the International Tooth Crown Company, we now come to the consideration of more recent patents, in which you are all vitally interested; viz. the Taggart patents for casting gold fillings and the like. This invention has revolutionized the practice of dentistry more than any other thing in my lifetime, and I am happy, as president of the Dental Protective Association to be able to endorse what Dr. Taggart has done and recognize his inventions on their merits. After careful study and investigation of his different patent claims, I believe they will stand the test, and will be validated by the Federal court when the proper time comes; and having come to this conclusion it behooved me, on the part of the Dental Protective Association, to get Dr. Taggart into an agreement before his patents were validated, thus guarding our members from any possible abuse by the owners of these patents, should they leave Taggart's hands, and prevent the possibility of exorbitant royalties being exacted. With this end in view, after long and strenuous efforts, the present Board of Directors of the Dental Protective Association consisting of Dr. C. N. Johnson, Dr. J. P. Buckley and myself, succeeded in effecting an agreement with Taggart and his attorneys which will absolutely protect the members of the association, both present and prospective. This agreement is a compromise measure and will certainly prevent a long line of litigation, and at the same time it is just and fair to both the present members of the Dental Protective Association and Dr. Taggart, and also to all members of the profession who desire to join the association.

Such an agreement as we succeeded in making with Dr. Taggart could not have been made with him on so favorable terms after he had his patents validated. Immediately after this agreement was obtained a circular was issued announcing the terms and the conditions upon which the profession could take advantage of it. Whether this agreement will accomplish what we anticipated will depend upon the individual members of the profession. After this circular was mailed many of the older members sent in their check for the required amount at once. Others hesitated because they did not seem to understand just what the Dental Protective Association was. In issuing this circular we overlooked the fact that fully four-fifths of the practicing dentists of today know practically nothing of what the Dental Protective Association has done and how much they individually have been benefited by its existence. When we stop to consider that patent litigation has been practically at a standstill for the past ten or fifteen years, it is not strange that the younger men of the profession should be ignorant of the work of the association. For this reason, in the first part of this paper, I have given you a brief history of the Dental Protective Association, showing how we put the International Tooth Crown Company out of business and prevented much patent abuse. You, therefore, have a faint idea of what you are being saved today in royalties because of this association. There is not a man practicing dentistry today in this country who is not being saved hundreds of dollars each year which he would have had to pay had these greedy corporations been left to their own designs.

There is one very important factor which I wish to discuss in this connection, and it is this: Not more than one-sixth of the dental profession came into the Dental Protective Association or contributed to the expense of this vast amount of litigation, yet the remaining five-sixths received the protection and benefits without paying a dollar. The only contributions many of these outsiders have made or are making today is their criticism of the association, mainly adverse and unfair.

When this Taggart proposition came up, and I had examined his patents and the evidence which will be presented to the Federal court, I studied it over carefully and came to the conclusion that Taggart would ultimately establish the validity of his patents. I felt that here was an opportunity for doing double duty. First, if an agreement could be effected it would be possible for Dr. Taggart to get a just revenue without court fights and without working a hardship on any man; and, secondly, it would place the Dental Protective Association on a strong and permanent basis. I knew that these objects could be accomplished only by means of a compromise agreement, making the terms so reasonable and fair to all parties concerned that each member of the profession would willingly join the Dental Protective Association to get the benefits thereof.

There are supposed to be about 40,000 dentists using Dr. Taggart's inventions in the United States alone. If this number came into the association, it is easy to see that a large fund of money would thus be created with a very light tax on each one. The income of such a fund, if properly invested, would take care of every form of abuse that might arise in the future and threaten the profession, and thus the permanency and efficiency of the Dental Protective Association would be assured.

One feature of this agreement is that to get the benefit of it, a dentist must be a member of the Dental Protective Association, and because of this fact, I have been accused of coercing or trying to coerce members of the profession into the association. Any dentist who feels thus loses sight of the fact that were it not for the association no such agreement could have been made with Dr. Taggart, for it was not a voluntary act on his part, and it now seems only just that the members of the profession who have been reaping the benefits of the association without contributing to its support, should come forward and do their part in this great work. If they do not, and let me state plainly here that there is absolutely no compulsion about the matter, but those who do not choose to join with us must stand, so far as the Dental Protective Association is concerned, on their own responsibility; and if sued by Dr. Taggart and forced by law to pay what might seem to be an unjust amount, they have only themselves to blame. This paper is presented at this time that every member of the profession may have knowledge of the fact that through the Dental Protective Association's agreement with Dr. Taggart they may have the advantage of all of Dr. Taggart's inventions without paying unjust or exorbitant royalties, unless it can be construed that the payment of 88 cents a year in advance for seventeen years, the life of the patents, for this privilege is unjust and exorbitant.

This agreement provides first, that any member of the association in good standing can secure the present Taggart machine for the cash sum of seventy-five dollars ($75.00) including the permission to use the method, providing the amount is paid within one year from the date of the entry of any decree or judgment finding Taggart's patents valid or granting damages for infringement, or he can obtain permission to practice the Taggart method alone for the lifetime of the patents (17 years) with any machine he may now be using for the cash sum of fifteen ($15.00) dollars, providing the amount is paid before the entry of any decree or judgment finding Taggart's patents valid or granting damages for infringement.

The agreement further provides that any member of the profession who joins the association within the time above specified can also secure such permission on the same terms.

The fee for membership in the association is ten ($10.00) dollars. It may be seen that for twenty-five ($25.00) dollars any member of the profession can obtain membership in the association and the permission to use the Taggart method. We feel that this is a fair and just proposition for all concerned, and we earnestly hope that all of the members of the association, both present and prospective, will accept the terms of the agreement.

Those who know anything about Federal court proceedings will appreciate the fact, that it may be only one month or it may be four or five years before Taggart's case comes up in Court for final adjudication and the members of the profession must understand that there is nothing to prevent Dr. Taggart and his attorneys from bringing suits against dentists who are using the methods with infringing machines without first obtaining the right to do so, either directly from him or through the Dental Protective Association. On this point there seems to be a great deal of misunderstanding. The profession is in error in thinking they need not take action until Taggart has validated his patents. There is nothing to hinder his lawyers from bringing as many suits as are advantageous to their cause at any time.

I wish to emphasize distinctly here that any member of the profession who is sued by Taggart cannot thereafter take advantage of the agreement, for the Protective Association is barred from furnishing them any assistance in the way of defense and they will be obliged to deal with Taggart and his lawyers independent of the association. Some such suits have already been begun, one right here in Chicago, where the party sued had to go and settle with Taggart and his lawyers for a much larger sum of money than the sum specified in this agreement.

As this agreement was a compromise measure it would be much easier to comply with this than to take the chance of being sued, and it is hoped that the members of this society will set the example and comply with this agreement as a body.

There is another mistaken idea which seems to cling to many members of the profession, and that is, that process patents cannot or should not be valid. Many are asking why, since I fought and did away with certain process patents in the past, do I not take this process patent of Taggart's and treat it similarly. What is to hinder other process patents being thrust upon us, they ask? These questioners lose sight of the fact, that there are patents claims which in the opinion of good judges will stand the test in Court because they are legitimate and based on the well recognized principle that for a thing to be patentable, it must be new and useful. Let me ask, What is a process patent? I can scarcely think of a patent that does not involve a process. What constitutes a valid patent then? There is no more well established principle relating to patent law that that the thing patented to be valid must be new and useful. The essential elements of utility and usefulness are the basis of our whole patent system and the statutory enactments thereunder. It is often difficult, and at times impossible, for the patent office to determine simply from an examination of the thing presented for a patent grant whether such thing is a new and useful improvement. As a matter of fact the patent office is unwilling to assume the responsibility of determining whether the elements of newness, usefulness and improvement in the thing presented actually exists. Therefore, many patents are granted which are neither new or useful and must come under the head of worthless patents. It was largely such patents as these which, in the past annoyed the dental profession.

You may ask, Is Taggart's invention new and useful? Some men claim to have done these things before Taggart. Granting for sake of argument that they did, which I am certain in my own mind they did not, let me ask, did these men give the ideas to us so that we might make use of them? Is there any man present who really doubts but that for Dr. Taggart we would still be filling teeth in the old way? Is there anyone here who is willing to give up the method given us by Taggart in every detail, and practice as he did before? I do not believe there is a man here tonight who would willingly give up the use of Taggart's method and fill teeth as he did before it was known for one hundred times what Taggart has offered to accept by the terms of the Dental Protective Association's agreement.

In answer to those who seem to lack confidence in the ability of the officers of the Dental Protective Association to determine valuable from worthless patents, and fear that the latter might be recognized in the future, let me say this: If anyone presents a patent based on the principle of being new and useful, they will be given due consideration and an opportunity offered for treating the members of the Dental Protective Association fairly. This is just what we did with Taggart, and let me say to his credit, that he was willing to be fair. What more could we ask? The association in the future, as in the past, will endeavor to protect its members, but its members only, from abuse. If this can be done by compromise, all well and good; if not, the association will exhaust its funds in attempting to protect its members. This is one of the strongest arguments for the existence of such a fund as we hope to create, for no sane man or group of men would attempt to abuse the members of any organization if he or they knew that a large sum of money was ready to be expended if necessary to prevent such abuse.

Since announcing to the profession the terms of the agreement with Taggart, I have been accused of not doing my duty because I did not fight Taggart and try to invalidate his patents. Why should I have fought Taggart when he was willing to treat the members of the Dental Protective Association fairly? I have always posed as an honest man and no honest man would fight Taggart under the circumstances. Surely, I would not as long as he attempted no abuse of the members of the association. Some, who do not understand the situation and who seem more willing to criticise than to put their shoulder to the wheel and help, say that the Dental Protective Association was organized to fight all patents, good, bad and indifferent. Since I organized this association myself, I ought to know the objects of its organization, and I know of no better way of stating this to you than to quote from the second circular that was issued to the profession in June 1889, over twenty-one years ago. In this circular I said:

"Its first object, as stated in the circular is to defend the profession against the unjust demands of patentees whose claims are worthless. Let it be distinctly understood that it is not the design of the Dental Protective Association to interfere with any man's legitimate business or valid patents, but to stop the enormous abuse of dental patents.

"Its second object is to bind the dental profession together for mutual protection, strength and helpfulness, with a bank account and without politics.

"An organization for defense must necessarily differ widely from one for social or political purposes. It must be ready for war at any moment. To insure this, the authority needs to be in the hands of a few. Such an organization is the Dental Protective Association. The power is invested in the directors: the responsibilities are borne by them, and all risks as well. They can sue or be sued, are accountable for the proper handling of the funds, and must take charge of the suit of any member of the association who is unjustly sued for infringement. Every member by paying a fee of ten ($10.00) dollars and assuming a liability of ten more, only (which latter will probably not be needed) can continue his practice undisturbed, knowing that if sued, he will be furnished with the best of legal talent and evidence, and be relieved of all costs and harrassment of suit. It will at once be seen that the largest expenditure of the association must be that of time, energy and thought on the part of the directors, since there are no salaried officers, and expenses are kept at the minimum.

"The first and greatest adversary, though not the only one, with which we have to deal, is the International Tooth Crown Company. Do you know how nearly you have been in its jaws? Look at a list of its patents. On bridgework it has several, including the Low, the Richmond, and the Sheffield bridges. It has a patent on preparing roots for crowns, which includes a patent on freezing the tooth, a patent on cutting off the tooth, a patent on killing the pulp, and on driving it out at the same time (if you can), a patent on filling the end of the root, and a patent on filling the root with material suitable for holding the metallic pin or screw which supports the crown or bridge.

"It had even a patent on the cement for securing crowns. On crowns it has many patents, including the Beers or gold crown, the Bitner crown, the Richmond crowns and the Sheffield crowns; several of each of the last two named. It has patents on crowns with bands and without bands; patents on crowns secured with screws and without screws; a patent on crowns secured with gutta percha, a patent on crowns secured with cement and a patent on crowns secured with both gutta percha and cement. It has also a patent on crowns covering the end of roots. In fact, if the validity of these various patents owned by the International Tooth Crown Company should be established, it would seem as if all the other crowns now in use would be declared an infringement on that company's crown patents. Then this company has also a patent on use of trial plates, a patent on getting articulating models, and a patent on investing the piece for soldering."

I quote the above simply to show that the accusations against me at this time are absolutely incorrect. I have uttered these sentiments time and time again during the past twenty years, to wit: *that the Dental Protective Association was not organized to deprive a man of his rights*, and in this instance after examining the patents which Dr. Taggart now holds, I am more and more convinced that the Court will in due time validate them; and in all the discussions I have heard, I have had but two men say he was not entitled to at least the small sum of fifteen ($15.00) dollars as compensation.

In this paper I have abstained from discussing the question of ethics in relation to patents or the propriety or wisdom of dental inventors taking out patents, except to say that there is no reason why an inventor should not be compensated for his inventions, and the only way for him to get compensation is to patent his discoveries, and the proof of this assertion is well illustrated in the present case. In this connection, I wish to call attention to a very able article in the September number of the *Dental Review*, by

Dr. Edmund Noyes, wherein the subject is treated exhaustively and ably and which should be read by every dentist.

Ever since the organization of the Dental Protective Association, I have at various times been accused of all sorts of unwarrantable acts. I have been criticized alike by the companies which I was fighting, and by many of the members of the profession on whose behalf I was making the fight. Sometimes I have answered the criticisms and sometimes I have not, because I have felt confident at all times that my acts would eventually be judged by the results, and I was willing to stand or fall by this test. Then, again, I knew that much of the criticism was based on a misunderstanding. This I feel is especially true of the agreement in the Taggart case, and my present plea is that men will look carefully into the merits in question before passing judgment. I have never claimed to be beyond the possibility of mistakes, because to be that I would be something more than human: and yet in this Taggart agreement I have had ample time to think it over, and I do not believe that any mistake has been made. In any event, I am willing to await the verdict of time and experience, and I have little doubt of the ultimate endorsement by my profession of the position I have taken.

In order to give something like a comprehensive review of the past and present patent litigations, it has been necessary for me to omit the discussion of patents on celluloid or aluminum and many other unimportant patents, as this paper is already unusually long, I hope you have not been wearied to such an extent that you cannot enter into a thorough and earnest discussion of the questions involved. I thank you for your patience.

